# A Reappraisal of the S2 State of Nature’s Water Oxidizing Complex in Its Low and High Spin Forms

**DOI:** 10.1021/acs.jpclett.4c00997

**Published:** 2024-05-28

**Authors:** Maxim Barchenko, Patrick J. O’Malley

**Affiliations:** Department of Chemistry, School of Natural Sciences, The University of Manchester, Manchester M13 9PL, United Kingdom

## Abstract

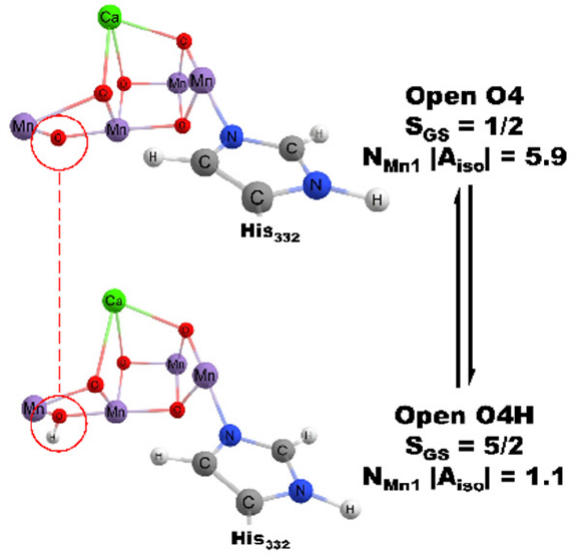

Density functional theory calculated ^14^N hyperfine couplings are obtained for the Mn1 ligated π-N of residue His332 of the photosystem 2 water oxidizing complex. An open cubane, O4H, model closely matches the experimental coupling obtained for the high spin *S* = 5/2 form of the S_2_ state, supporting an open cubane structure for this state. We also investigate the unusual geometric features for the S_2_ state obtained by X-ray free electron laser structure determinations and rationalize it as an equilibrium occurring at room temperature between W1/O4 deprotonated and protonated forms of the open cubane structure.

Water oxidation in nature is performed in the water oxidizing complex (WOC) of photosystem 2.^1^ To achieve this key evolutionary step in biology, Kok^2^ suggested that the four photons of visible light energy progressively oxidize the complexed water molecules evolving molecular oxygen on the fourth photon. The five oxidized states are designated as S_*n*_, where *n* = 0–4. To understand the water oxidation mechanism, it is necessary to obtain the geometric and electronic structure of each of these states. The S_2_ state is probably one of the best characterized states. Electron paramagnetic resonance (EPR) spectroscopy^3,4^ has shown that, for this S_2_ state, an *S* = 1/2 low spin (LS) ground state characterized by a *g* = 2 multiline signal or an additional broad featureless signal at *g* = 4.1 is observed depending on sample preparation. The *g* = 4.1 form has been shown to be an *S* = 5/2, high spin (HS) system. For cyanobacteria, high pH has been shown to lead to a high spin form of S_2_ having an increased *g* value of 4.8.^5^ Despite the varying *g* values, this high pH form was also assumed to correspond to an *S* = 5/2 species with similar origin as the *g* = 4.1 signal. A recent report suggested a different structural origin for the *g* = 4.1 and *g* = 4.8 signals.^6^ This is unlikely however as it has been demonstrated by Boussac et al.^7^ that these two forms are interconvertible.

Pantazis et al.^8^ suggested that the two spin states of S_2_ corresponded to valence and structural isomers, with the LS state corresponding to an open cubane form, Figure 1, having Mn_1_(III) and Mn_2,3,4_(IV) while the HS state is a closed cubane form with Mn_4_(III) and Mn_1,2,3_(IV). Similar termed right-hand and left-hand cubane geometries were suggested by Yamaguchi et al.^9^ Bovi et al.^10^ later proposed that the closed cubane form was required to form the S_3_ state.

**Figure 1:**
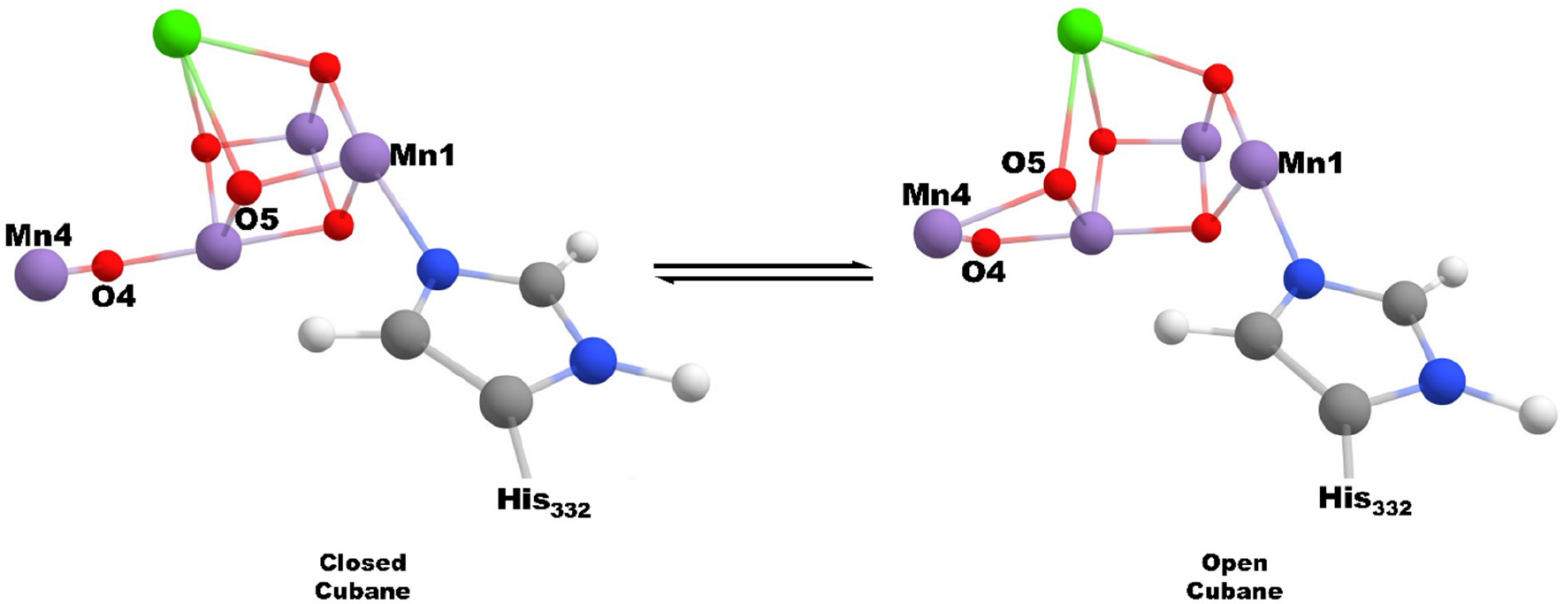
Open closed cubane equilibrium structures for a water oxidizing complex, including ligation of His332 to Mn1.

In recent years, therefore, the widely accepted consensus is to assign the LS and HS forms to open and closed cubane forms of the WOC, respectively (Figure 1). In addition, these respective forms are widely proposed to have a key role in the water oxidation reaction, leading to proposals such as the pivot and carousel mechanisms.^11,12^ However, a problem^13^ with the open and closed cubane equilibrium model for S_2_ is its inability to clearly rationalize how various treatments can preferentially stabilize one particular form as demonstrated experimentally using EPR.^14^ The XFEL crystal structure of the one-flash/1F state (mainly S_2_)^15^ shows an open cubane structure with no detection of a closed form as it progresses to S_3_. The lack of any structural evidence for a closed cubane form brings into question the necessity for a significant structural change to a closed cubane form to generate an *S* = 5/2 or higher spin state. Alternatively, we have previously reported broken symmetry density functional (BS-DFT) calculations on S_2_ open cubane cluster models^13^ with O4 modeled as either a bridging μ-oxo or a μ-hydroxo ligand between Mn_4_ and Mn_3_. A Heisenberg–Dirac–van Vleck (HDvV) spin ladder analysis was shown to give an *S* = 5/2 ground spin state for the O4 protonated open cubane form, showing that protonation of O4 can cause a switch between LS and HS forms without invoking an open to closed cubane transformation. We also showed that the spin state of the O4 protonated form switches from an *S* = 5/2, *g* = 4.1 ground state for models containing the W1Mn_4_ ligand as a water molecule to an *S* = 7/2, *g* = 4.8 ground state for W1 as a hydroxo.^17^ Pushkar et al.^16^ have also proposed early binding of a water molecule to explain this high spin state. Despite these alternatives and the lack of any direct structural support, the closed cubane model is still widely proposed as an intermediate not only in the S_2_ to S_3_ transition but has recently been proposed based on molecular modeling to be present in all S state transitions of the Kok cycle.^18^

In terms of experimental evidence supporting a closed cubane form, the original Pantazis et al. interpretation of the *S* = 5/2 ground state as corresponding to a closed cubane form remains the sole exhibit. In a recent report,^19^ ESEEM studies showed that the ^14^N hfc of WOC Mn1 ligand His332 for the *g* = 4.1 HS form has a magnitude of around 1 MHz and was significantly reduced compared with the LS form where a value of near 7 MHz has been reported previously.^20^ This important new piece of information was rationalized as strong support for the closed cubane form and to rule out an open cubane WOC origin for the HS *g* = 5/2 EPR signal.^19^ To further clarify this assessment, we now report BS-DFT calculated ^14^N hfc for this nucleus in an open cubane O4/O4H and a closed cubane model. We demonstrate that the magnitude of the experimental ^14^N hfc is in fact fully supportive of an open cubane WOC with O4 protonated. We also investigate the recently highlighted^21^ anomalies between model compound calculated geometry for the S_2_ state and that obtained by XFEL structure determination and rationalize that the major differences can be explained by an equilibrium occurring at room temperature between the O4 deprotonated and protonated forms of the open cubane structure.

The geometries for the chosen models were optimized in the ferromagnetic state, and then ground spin state and spin projection coefficients were determined with BS-DFT and Heisenberg–Dirac–van Vleck (HDvV) spin ladder calculations. EPR calculations were conducted for the ground spin state as determined by the spin ladder, and hyperfine couplings were corrected by a factor of the spin projection coefficients. Further details about the computational method are accessible in the Supporting Information. Calculated ^14^N hfc values for open cubane O4, open cubane O4H, and closed cubane models are given in Table 1. The open cubane deprotonated O4 model has a ground spin state of *S* = 1/2 (BS-DFT Mn centers αββα) and a ^14^N hfc of 5.9 MHz, in good agreement with the LS experimental value of 7 MHz. Protonation of O4 results in a shift of the ground spin state to *S* = 5/2 (BS-DFT Mn centers βααα), alongside considerable reduction in the magnitude of the calculated coupling to 1 MHz, in excellent agreement with the experimental determination. The oxidation states of all Mn centers remain consistent between the open cubane models, with Mn_1_(III) and Mn_2,3,4_(IV). It is therefore quite apparent that, in contrast to the conclusions recently reported, the experimentally determined ^14^N hfc of His332, for the HS form, strongly supports rather than rules out an open cubane form for the HS S_2_ state.

**Table 1:** BS-DFT Calculated and Experimentally Determined Isotropic Hyperfine Couplings for ^14^N of His332-Mn1

model	π-^14^N-His332 isotropic hfc/MHz
calculated	
open cubane O4	–5.91
open cubane O4H	1.05
closed cubane	–1.07
experimental magnitude^19,20^	
LS	7
HS	1

The recent high resolution XFEL structure of the single flash (mainly S_2_), 1F/S_2_, state clearly shows only the presence of the open cubane form, with no evidence of the closed cubane form.^15^ The open cubane form is retained as well in time-resolved points progressing to the next 2F/S_3_ state, ruling out any closed cubane intermediate occurring in this transition. Of relevance is that the 1F/S2 structure coordinates reveal a particularly short O4—OW19 distance of 2.4 Å for the 1F structure and, as emphasized in ref (15), a considerable decrease from the 0F state value of 2.7 Å. In addition, a neighboring hydrogen-bonded water molecule W20 present in 0F/S_1_ states is not detected in 1F/S_2_. The shortening of this distance is triggered by the oxidation of Mn_4_(III) to Mn_4_(IV) in the 1F/S_2_ state. To probe this further, we have calculated this distance for WOC models where O4 acts as a hydrogen bond donor (O4H) and as a hydrogen bond acceptor O4 to W19, Figure 2.

**Figure 2:**
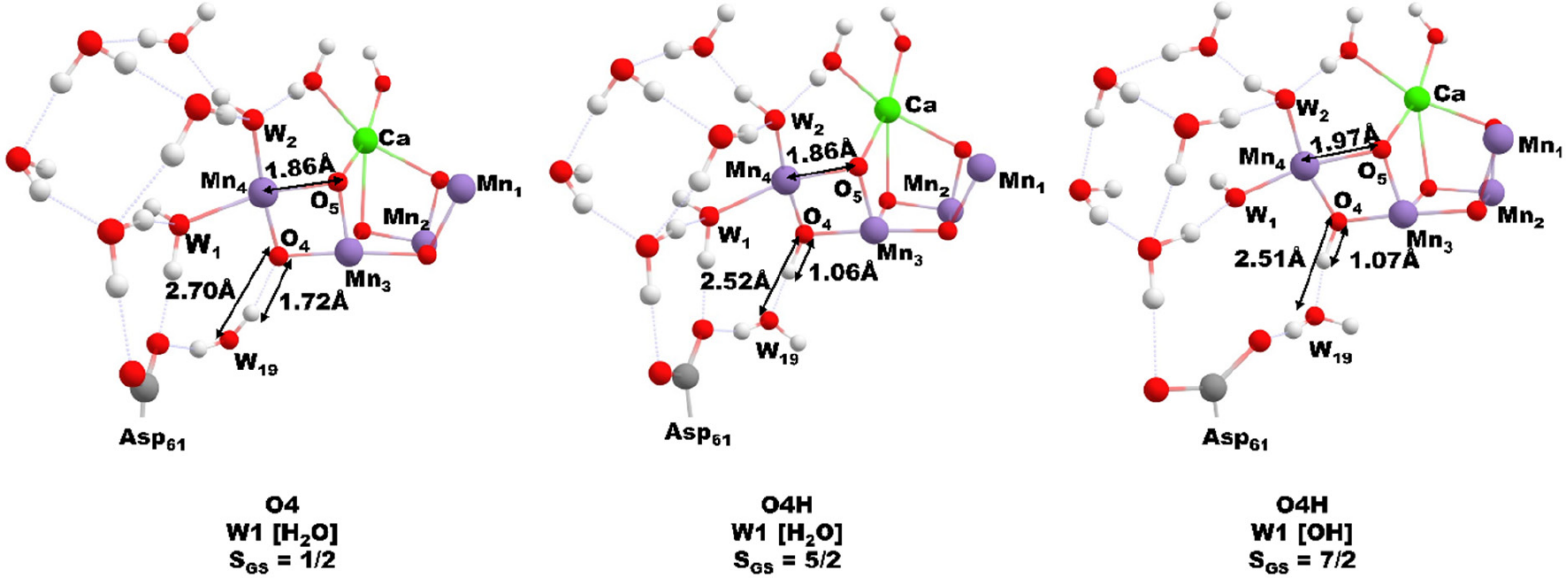
Equilibrium model between HS and LS forms of the S_2_ state, with key calculated bond distances given.

From our model calculations, there is a significant change in the O4—OW19 bond distance associated with this. With the role of O4 as a proton donor, the O4—OW19 bond distance is reduced by around 0.2 Å compared with when it accepts a hydrogen bond from W19. The significant directional strength of the hydrogen bond for the O4 donating form is illustrated in the IBOs for this bond shown in Figure 3 where the increased hydrogen bonding strength can be attributed to the strong overlap between the p type lone pair of the W19 water oxygen and the O4 proton. Such an interaction is not feasible when O4 acts as a hydrogen bond acceptor.

**Figure 3:**
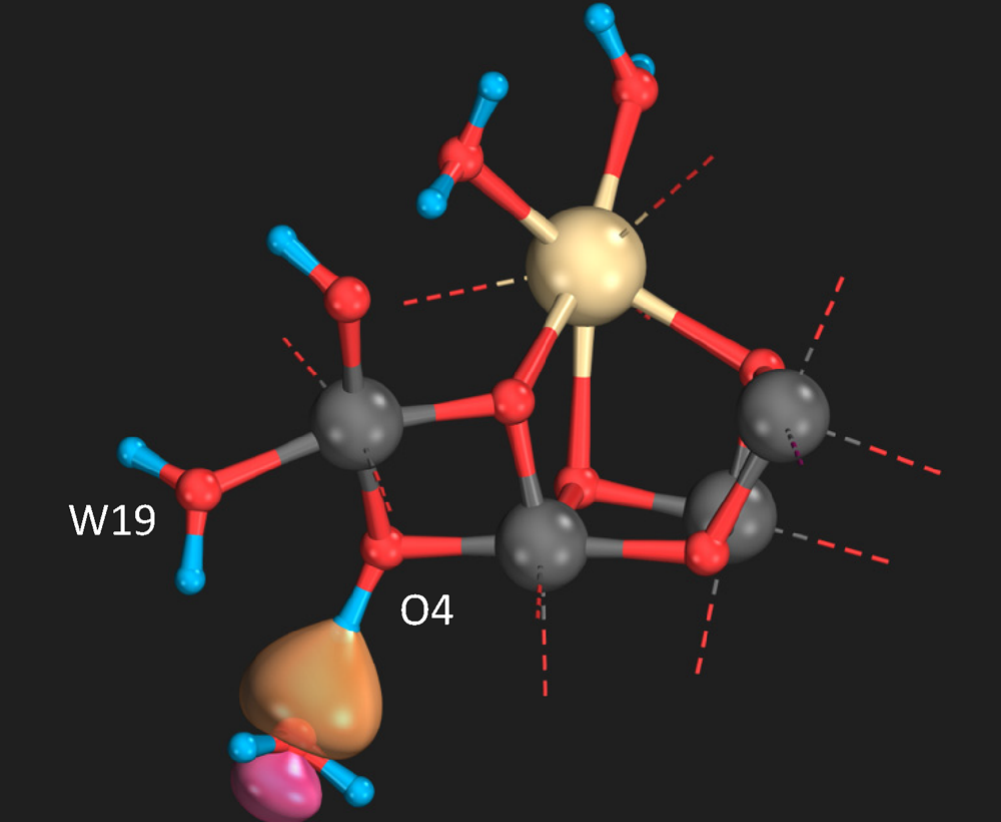
Lone pair p-type intrinsic bond orbital of W19 oxygen demonstrating strong overlap with the proton of the O4 when O4 acts as proton donor.

The stronger hydrogen-bond between O4 and OW19 due to hydrogen-bond donation by O4 together with the change in hydrogen-bonding nature would disturb/weaken the hydrogen-bonding environment of W20 and be expected to lead to release of W20 or increased flexibility/mobility, in line with the XFEL data interpretation. Another notable feature of the S_2_ state XFEL structure is a larger than expected Mn4–O5 bond distance of 2.0–2.2 Å. As was recently pointed out, this is in disagreement with what is expected from computational S_2_ open cubane models where a bond distance of 1.8 Å is calculated.^21^ Most of the previous S_2_ state models have been formed with W1 as an aquo ligand.^21^ When W1 is a hydroxo group, however, the Mn4–O5 bond has been shown^22^ to extend by 0.2–0.3 Å, close to that reported in the XFEL structure determinations. This is well demonstrated by the HS O4H model with W1 as the OH where the Mn4–O5 bond length is extended to 2.0 Å, Figure 2. This can be attributed to the greater trans effect of the OH ligand, which weakens the Mn4–O5 bond strength. Such a feature leading to a weakened Mn4–O5 bond would be expected to render O5 more open to bonding, with the extra O6 appearing in the S_3_ state. Experimental evidence supporting this scenario comes from the oxygen isotope exchange experiments reported by de Lichtenberg and Messinger.^23^ There, they showed that slow exchanging water has an enhanced exchange rate for high pH samples. The slow exchanging species is usually assigned to O5. This is in accord with a weakened Mn4O5 bond for such samples due to the presence of a W1 OH group present at high pH. Both of these key structural features present in the 1F XFEL structure determination are fully supportive of an open cubane WOC with a protonated O4 and W1 as a hydroxo ligand. This was the model we previously proposed to represent the *g* = 4.8–4.9 EPR detected form for samples poised at high pH values.^17^ At neutral pH, the multiline signal characteristic for an *S* = 1/2 LS form is principally detected. In this respect, it is important to note that the EPR measurements of the S_2_ state are performed at cryogenic temperatures in contrast to the room temperature XFEL structure determinations. If, as proposed above, the S_2_ form measured using XFEL at room temperature corresponds to an equilibrium mixture of protonated/deprotonated O4 and W1 forms, then EPR data can be explained by a shifting of this equilibrium at cryogenic temperatures. Thus, at neutral pH, the LS multiline signal may be caused by the predominance of the deprotonated O4/aquo W1 forms, while high pH may result in irreversible deprotonation of W1 to a hydroxo, which is then observed at low temperatures via EPR as the HS *S*_GS_ = 7/2, *g* = 4.8 form. Deprotonation of W1 is likely favored by the strong hydrogen bond with the Asp61 residue facilitating proton removal via the nearby water channel. For O4, a pathway to deprotonation is prevented by the absence of nearby W20 in the 1F/S2 state, disrupting proton removal via the water channel.

In conclusion, comparison of density functional theory calculated ^14^N hyperfine couplings for the π-N of residue His332 of the photosystem 2 water oxidizing complex with experimental values reveals that an open cubane, the model of O4H, closely matches the experimental coupling obtained for the high spin *S* = 5/2 form of the S_2_ state. This supports an open cubane structure for this state, in contrast to previous conclusions. The geometry for the S_2_ state obtained by X-ray free electron laser structure determinations is best rationalized as an equilibrium occurring at room temperature between W1/O4 deprotonated and protonated forms of the open cubane structure.

## Supporting Information Available

Details of methodologies for calculations performed and models used, with Mulliken spin populations of the presented models, and Cartesian coordinates for all optimized structures (PDF)
