# Supplementary material for: A Reappraisal of the S2 State of Nature’s Water Oxidizing Complex in Its Low and High Spin Forms
Source: J Phys Chem Lett. 2024 May 28;15(22):5883–6. doi: 10.1021/acs.jpclett.4c00997 (PMC11163464; doi:10.1021/acs.jpclett.4c00997)
Supplement: Supplementary file 1 — jz4c00997_si_001.pdf [file jz4c00997_si_001.pdf]

Supporting Information:

A Reappraisal of the S2 State of Nature's Water Oxidizing Complex in its Low and High Spin forms

Maxim Barchenko and Patrick J. O'Malley*

Department of Chemistry, School of Natural Sciences, The University of Manchester, Manchester, M13 9PL, UK.

METHODS

The detailed computational procedure used has been described previously in detail.[1–3] All calculations were performed using ORCA 4[4] with models initially geometry optimised in the fully ferromagnetic state.[1] Optimisation calculations used the BP86 functional[5,6] with the zeroth-order regular approximation (ZORA) Hamiltonian applied to include scalar relativistic effects.[7–9] ZORA versions of the def2-SVP basis sets were used for C and H atoms. ZORA versions of the def2-TZVP basis set with f functions removed were used for all other atoms.[10] The resolution of identity approximation (RI-J) for coulomb integrals, along with the decontracted general Weigend auxiliary basis sets were used.[11,12] Dispersion corrections proposed by Grimme with Becke-Johnson damping (D3BJ) were included.[13,14] The conductor-like polarizable continuum model (CPCM) with a dielectric constant $\varepsilon = 8.0$[15,16] was applied in all calculations. Increased integration grids (Grid6 and IntAcc 6 in ORCA convention) and tight SCF convergence criteria were used. All terminal carbon atoms of the modelled amino acid residues were constrained during optimisation calculations. The hybrid

meta-GGA TPSSh functional[17] was used for BS-DFT[18] and EPR calculations. The chain of spheres (RIJCOSX) approximation to exact exchange was applied along with the same decontracted auxillary basis sets involved in geometry optimization calculations.[19] ZORA versions of the def2-TZVP basis set with f functions removed was used for all atoms. Initial BS guesses were constructed using the 'flipspin' feature of ORCA.[4] Convergence to the correct BS and HS states in all calculations was confirmed by examination of the calculated Mulliken spin populations. The 'orca_eca' module within ORCA was used to calculate HDvV spin ladders and spin projection coefficients.[20] Calculation of the hfcs used the def2-TZVP basis set modified by inclusion of fully decontracted s-shells with three additional steep primitives added to the core for Mn, O, and N atoms; the def2-TZVP(-f) basis set was used for all other atoms.[4,21]

Model Systems

The models were generated from the S_2 XFEL crystal structure (PDB: 6DHF)[22] and optimized into the S_2 state as described in computational details. The models include directly coordinated (Asp-170, Glu-189, His-332, Glu-333, Asp-342, Ala-344, Glu-354) and second sphere (Asp-61, Tyr-161, Gln-165, Ser-169, His-190, Asn-298, His-337, Arg-357, alongside the partial backbone of Glu-329) amino acid residues. Water molecules are also included in the models, two coordinated on Mn_4 (W1 and W2), two coordinated on Ca (W3 and W4), and sixteen crystallographic waters. The differences between models discussed in this work are otherwise explained in their names and labels – closed and open cubane models differ as depicted in figure 1, O4 acts as either a μ-oxo or μ-hydroxo ligand (O4/O4H respectively), and W1/W2 can be either H_2O or ^-OH ligands.

Table S1 – BS-DFT Mulliken Spin Populations of Mn centers for each model presented in Table 1 and Figure 2.

Model	Mn1	Mn2	Mn3	Mn4
**Open O4 W1[H_2O] W2[OH]**<br><br>**(Table 1 – Open Cubane O4, Figure 2 – Left)**	3.96	-2.92	-2.83	2.94
**Open O4H W1[H_2O] W2[OH]**<br><br>**(Table 1 – Open Cubane O4H, Figure 2 – Mid)**	-3.96	2.94	2.89	2.90
**Open O4H W1[OH] W2[OH]**<br><br>**(Figure 2 – Right)**	-3.96	2.92	2.87	2.94
**Closed O4 W1[H_2O] W2[OH]**<br><br>**(Table 1 – Closed Cubane)**	2.97	2.91	2.78	-3.90

REFERENCES

(1) Corry, T. A.; O'Malley, P. J. Proton Isomers Rationalize the High- and Low-Spin Forms of the S 2 State Intermediate in the Water-Oxidizing Reaction of Photosystem II. *J. Phys. Chem. Lett.* **2019**, *10* (17), 5226–5230.

(2) Corry, T. A.; O'Malley, P. J. Molecular Identification of a High-Spin Deprotonated Intermediate during the S2 to S3 Transition of Nature's Water-Oxidizing Complex. *J. Am. Chem. Soc.* **2020**, *142* (23), 10240–10243.

(3) Corry, T. A.; O'Malley, P. J. Evidence of O−O Bond Formation in the Final Metastable S3 State of Nature's Water Oxidizing Complex Implying a Novel Mechanism of Water Oxidation. *J. Phys. Chem. Lett.* **2018**, *9* (21), 6269−6274.

(4) Neese, F. Software Update: The ORCA Program System, Version 4.0. *Wiley Interdiscip. Rev.: Comput. Mol. Sci.* **2018**, *8* (1), e1327.

(5) Perdew, J. Density-Functional Approximation for the Correlation Energy of the Inhomogeneous Electron Gas. *Phys. Rev. B: Condens. Matter Mater. Phys.* **1986**, *33* (12), 8822−8824.

(6) Becke, A. D. Density-Functional Exchange-Energy Approximation with Correct Asymptotic Behavior. *Phys. Rev. A: At., Mol., Opt. Phys.* **1988**, *38* (6), 3098−3100

(7) van Lenthe, E.; Baerends, E. J.; Snijders, J. G. Relativistic Regular Two-Component Hamiltonians. *J. Chem. Phys.* **1993**, *99* (6), 4597

(8) van Lenthe, E.; Baerends, E. J.; Snijders, J. G. Relativistic Total Energy Using Regular Approximations. *J. Chem. Phys.* **1994**, *101* (11), 9783

(9) van Wüllen, C. Molecular Density Functional Calculations in the Regular Relativistic Approximation: Method, Application to Coinage Metal Diatomics, Hydrides, Fluorides and Chlorides, and Comparison with First-Order Relativistic Calculations. *J. Chem. Phys.* **1998**, *109* (2), 392.

(10) Weigend, F.; Ahlrichs, R. Balanced Basis Sets of Split Valence, Triple Zeta Valence and Quadruple Zeta Valence Quality for H to Rn: Design and Assessment of Accuracy. *Phys. Chem. Chem. Phys.* **2005**, *7* (18), 3297.

(11) Eichkorn, K.; Treutler, O.; Öhm, H.; Häser, M.; Ahlrichs, R. Auxiliary Basis Sets to Approximate Coulomb Potentials. *Chem. Phys. Lett.* **1995**, *240* (4), 283−289

(12) Weigend, F. Accurate Coulomb-Fitting Basis Sets for H to Rn. *Phys. Chem. Chem. Phys.* **2006**, *8* (9), 1057−1065.

(13) Grimme, S.; Antony, J.; Ehrlich, S.; Krieg, H. A Consistent and Accurate Ab Initio Parametrization of Density Functional Dispersion Correction (DFT-D) for the 94 Elements H-Pu. *J. Chem. Phys.* **2010**, *132* (15), 154104.

(14) Grimme, S.; Ehrlich, S.; Goerigk, L. Effect of the Damping Function in Dispersion Corrected Density Functional Theory. *J. Comput. Chem.* **2011**, *32* (7), 1456−1465.

(15) Barone, V.; Cossi, M. Quantum Calculation of Molecular Energies and Energy Gradients in Solution by a Conductor Solvent Model. *J. Phys. Chem. A.* **1998**, *102* (11), 1995-2001

(16) Pantazis, D. A.; Ames, W.; Cox, N.; Lubitz, W.; Neese, F. Two Interconvertible Structures That Explain the Spectroscopic Properties of the Oxygen-Evolving Complex of Photosystem II in the S2 State. *Angew. Chem. Int. Ed. Engl.* **2012**, *51* (39), 9935–9940.

(17) Staroverov, V. N.; Scuseria, G. E.; Tao, J.; Perdew, J. P. Comparative Assessment of a New Nonempirical Density Functional: Molecules and Hydrogen-Bonded Complexes. *J. Chem. Phys.* **2003**, *119* (23), 12129.

(18) Pantazis, D. A.; Orio, M.; Petrenko, T.; Zein, S.; Bill, E.; Lubitz, W.; Messinger, J.; Neese, F. A New Quantum Chemical Approach to the Magnetic Properties of Oligonuclear Transition-Metal Complexes: Application to a Model for the Tetranuclear Manganese Cluster of Photosystem II. *Chem. - Eur. J.* **2009**, *15* (20), 5108−5123

(19) Neese, F.; Wennmohs, F.; Hansen, A.; Becker, U. Efficient, Approximate and Parallel

Hartree-Fock and Hybrid DFT Calculations. A “chain-of-Spheres” Algorithm for the Hartree-Fock Exchange. *Chem. Phys.* **2009**, *356* (1−3), 98−109.

(20) Rapatskiy, L.; Ames, W.M.; Perez-Navarro, M.;, Savitsky, A.; Griese, J.J.; Weyhermuller, T.; Shafaat, H.S.; Hogbom, M.; Neese, F.; Pantazis, D.A.; Cox, N. Characterization of Oxygen Bridged Manganese Model Complexes Using Multifrequency ^{17}O-Hyperfine EPR Spectroscopies and Density Functional Theory. *J. Phys. Chem. B* **2015**, *119* (43) 13904-13921

(21) Cox, N.; Ames, W.; Epel, B.; Kulik, L.V.; Rapatskiy, L.; Neese, F.; Messinger, J.; Wieghardt, K.; Lubitz, W. Electronic Structure of a Weakly Antiferromagnetically Coupled $Mn^{II}Mn^{III}$ Model Relevant to Manganese Proteins: A Combined EPR, ^{55}Mn-ENDOR, and DFT Study. *Inorg. Chem.* **2011**, *50* (17), 8238-8251

(22) Kern, J.; Chatterjee, R.; Young, I. D.; Fuller, F. D.; Lassalle, L.; Ibrahim, M.; Gul, S.; Fransson, T.; Brewster, A. S.; Alonso-Mori, R.; et al. Structures of the Intermediates of Kok’s Photosynthetic Water Oxidation Clock. *Nature* **2018**, *563* (7731), 421–425.

Cartesian Coordinates of Optimized Structures

Open O4H W1[OH] W2[OH]

Ca	-32.687093000	39.761589000	362.035071000
O	-33.184926000	39.563992000	364.383797000
O	-31.669189000	37.757925000	363.231868000
O	-33.616091000	37.113410000	364.752883000
O	-32.348460000	35.784491000	361.423642000
O	-33.968640000	37.562223000	361.984248000
Mn	-34.767399000	38.629637000	364.633909000
Mn	-32.064108000	38.198345000	364.932844000
Mn	-32.988096000	36.477381000	363.021560000
Mn	-33.851566000	36.449623000	360.367165000
C	-30.033546000	34.448867000	365.303944000
C	-31.036033000	35.489099000	364.944813000
O	-31.020479000	36.611222000	365.570337000
O	-31.834471000	35.180442000	364.002171000
H	-29.220052000	34.488630000	364.555561000

H	-30.484947000	33.445162000	365.256950000
H	-29.593258000	34.621363000	366.298129000
C	-32.796152000	30.220033000	361.294719000
C	-33.841417000	31.246075000	360.853025000
O	-33.700134000	32.447971000	361.287183000
O	-34.795365000	30.891902000	360.094714000
H	-33.005574000	29.220248000	360.881941000
H	-32.772158000	30.163688000	362.397797000
H	-31.793729000	30.546481000	360.963387000
C	-29.073591000	44.563795000	354.927778000
C	-30.035013000	43.374710000	355.039385000
C	-30.960514000	43.384467000	356.246518000
C	-31.721286000	42.235833000	356.568301000
C	-31.113972000	44.510281000	357.080641000
C	-32.577396000	42.199315000	357.678678000
C	-31.970095000	44.493264000	358.193535000
C	-32.704001000	43.329442000	358.525481000
O	-33.482390000	43.279899000	359.633878000
H	-29.615553000	45.520095000	354.812966000
H	-30.650013000	43.318843000	354.117368000
H	-29.452379000	42.432057000	355.053684000
H	-31.633387000	41.343512000	355.931786000
H	-30.548971000	45.426956000	356.872169000
H	-33.148809000	41.295203000	357.927882000
H	-32.050702000	45.384067000	358.826776000
H	-33.519054000	44.496967000	360.393216000
H	-28.430442000	44.648562000	355.823527000
H	-28.414667000	44.449188000	354.049068000
C	-29.253922000	43.004351000	359.721419000
C	-29.685174000	44.209007000	360.521779000
N	-29.161150000	45.400809000	360.161918000
O	-30.484163000	44.113809000	361.485615000
H	-28.476265000	43.234519000	358.976924000
H	-30.135823000	42.595503000	359.196641000
H	-28.555676000	45.493203000	359.354098000
H	-29.446579000	46.246600000	360.647099000
H	-28.886729000	42.227757000	360.413523000
C	-30.088214000	34.401171000	356.344816000
C	-31.122067000	35.486611000	356.596214000
O	-32.345691000	35.217306000	356.698891000
H	-30.318690000	33.913969000	355.381227000
H	-30.175744000	33.633667000	357.133034000
H	-29.055526000	34.784586000	356.318563000
N	-30.656226000	36.752625000	356.689413000
C	-31.491625000	37.950793000	356.789409000
C	-31.165621000	38.801997000	358.017284000
C	-31.678005000	38.274605000	359.358492000
O	-31.232567000	38.747184000	360.436335000
O	-32.611951000	37.390315000	359.242381000
H	-29.655526000	36.890785000	356.572433000
H	-32.546211000	37.633084000	356.816340000
H	-30.081565000	38.997872000	358.110400000
H	-31.640618000	39.794611000	357.912838000
H	-31.335499000	38.556558000	355.876902000
C	-36.293781000	42.469506000	363.932042000
C	-35.631465000	41.169638000	363.549606000
O	-34.923356000	41.034018000	362.530747000

O	-35.876122000	40.190260000	364.391112000
H	-36.014095000	42.725324000	364.968162000
H	-37.391890000	42.366099000	363.872209000
H	-35.966234000	43.280850000	363.265876000
C	-36.037814000	48.310983000	361.463650000
C	-34.985668000	47.272351000	361.333593000
C	-35.011634000	46.029935000	360.731841000
N	-33.703556000	47.416491000	361.845385000
C	-32.983266000	46.308080000	361.573803000
N	-33.766694000	45.460279000	360.897641000
H	-36.940215000	48.000719000	360.913296000
H	-35.701241000	49.281319000	361.056583000
H	-35.810090000	45.518127000	360.195045000
H	-31.950833000	46.110279000	361.863149000
H	-36.321511000	48.474539000	362.519608000
C	-34.720683000	51.637782000	364.203879000
C	-34.280493000	50.197515000	363.972647000
N	-34.822040000	49.245214000	364.763850000
O	-33.453446000	49.906568000	363.070290000
H	-35.408307000	51.744341000	365.057518000
H	-35.215232000	52.005863000	363.288179000
H	-34.551267000	48.262824000	364.658443000
H	-35.489862000	49.480757000	365.490464000
H	-33.824977000	52.259467000	364.372582000
H	-33.401613000	48.267706000	362.372753000
C	-40.681662000	40.131027000	367.699860000
C	-40.753776000	38.762182000	368.309648000
O	-40.344147000	37.716943000	367.733517000
H	-41.359805000	40.173660000	366.828249000
N	-41.301933000	38.684113000	369.532078000
C	-41.433296000	37.412209000	370.206975000
H	-41.648286000	39.526903000	369.979008000
H	-40.447370000	36.930443000	370.330880000
C	-39.851593000	36.488341000	364.048019000
C	-38.550619000	36.951652000	364.585880000
C	-37.304868000	37.110068000	364.002070000
N	-38.401748000	37.341947000	365.907806000
C	-37.128886000	37.726385000	366.109894000
N	-36.432928000	37.594329000	364.967036000
H	-39.765293000	36.270944000	362.971223000
H	-40.636498000	37.255501000	364.179719000
H	-36.985620000	36.938510000	362.973139000
H	-36.736301000	38.128126000	367.042070000
H	-40.197787000	35.569494000	364.556148000
H	-40.961186000	40.927507000	368.407607000
H	-39.659128000	40.304188000	367.323288000
H	-41.877970000	37.579232000	371.199447000
H	-42.080356000	36.723370000	369.634925000
H	-39.181725000	37.471779000	366.607147000
C	-36.210088000	33.753083000	362.748233000
C	-35.186104000	34.780267000	362.363336000
O	-34.325495000	35.085362000	363.263314000
O	-35.217396000	35.277870000	361.191263000
H	-35.831558000	32.782676000	362.379163000
H	-37.172176000	33.955467000	362.251514000
H	-36.332834000	33.712381000	363.840193000
C	-33.613534000	34.102019000	370.372090000

C	-33.921138000	34.614371000	368.996759000
C	-33.239916000	35.468444000	368.142771000
N	-35.075186000	34.255528000	368.303583000
C	-35.106246000	34.848839000	367.096609000
N	-33.996979000	35.586985000	366.985768000
H	-34.406923000	34.386404000	371.086000000
H	-32.659731000	34.526353000	370.721800000
H	-32.289795000	35.996315000	368.269283000
H	-35.887878000	34.743108000	366.346310000
H	-33.782840000	36.167791000	366.131418000
H	-33.528895000	33.000670000	370.373042000
H	-35.800113000	33.630844000	368.651049000
C	-33.579444000	39.662687000	368.687086000
C	-33.597380000	39.037341000	367.329386000
O	-32.481050000	38.567900000	366.872669000
O	-34.691052000	39.023622000	366.692457000
H	-34.601732000	39.875302000	369.031128000
H	-33.020938000	40.614112000	368.617140000
H	-33.045362000	39.022745000	369.410410000
C	-29.204110000	41.144943000	365.018420000
C	-30.174850000	40.211083000	364.370619000
O	-30.571963000	40.357329000	363.193985000
O	-30.558590000	39.268934000	365.196127000
H	-29.789870000	41.844107000	365.644297000
H	-28.510084000	40.604517000	365.681771000
H	-28.650713000	41.730245000	364.267425000
C	-25.798943000	37.259595000	362.310142000
N	-27.074882000	36.572010000	362.084521000
C	-28.283924000	37.162873000	362.017830000
N	-28.405630000	38.489332000	362.190978000
N	-29.379417000	36.432664000	361.758960000
H	-25.763619000	37.723395000	363.312786000
H	-25.623169000	38.032930000	361.541185000
H	-27.051001000	35.565685000	361.957429000
H	-27.624069000	39.036727000	362.531469000
H	-29.327873000	38.928989000	362.188848000
H	-29.310518000	35.449203000	361.523018000
H	-30.311755000	36.854555000	361.784293000
H	-24.998781000	36.509371000	362.243756000
O	-31.476919000	33.459101000	361.766238000
H	-32.391165000	32.929554000	361.604074000
H	-31.453704000	33.599657000	362.736555000
O	-35.710167000	41.955332000	359.917205000
H	-34.880316000	42.517398000	359.724475000
H	-35.649870000	41.786818000	360.888616000
O	-33.715811000	35.105699000	359.131491000
H	-33.228960000	35.392959000	358.317869000
O	-32.308541000	42.110382000	361.656435000
H	-31.460041000	42.635892000	361.666654000
H	-32.778381000	42.481178000	360.837330000
O	-35.791330000	45.116940000	366.563491000
H	-35.134806000	45.500642000	365.902113000
H	-36.612670000	45.001696000	366.051950000
O	-35.204361000	37.427569000	359.591436000
H	-35.026274000	37.558835000	358.591709000
O	-35.511947000	33.195501000	358.758375000
H	-34.803127000	33.847463000	359.052138000

H	-35.327330000	32.370987000	359.306253000
O	-37.740176000	40.758929000	366.306133000
H	-38.129409000	41.584370000	365.963799000
H	-37.041517000	40.516499000	365.634305000
O	-37.183184000	35.464475000	358.880332000
H	-36.550701000	36.115758000	359.283702000
H	-36.708145000	34.596545000	358.987926000
O	-34.710482000	33.750934000	356.176429000
H	-34.981792000	33.409477000	357.077999000
H	-33.815883000	34.140055000	356.328841000
O	-36.435854000	40.450578000	357.589396000
H	-36.226866000	40.922760000	358.435347000
H	-36.202543000	41.098919000	356.899375000
O	-36.440290000	35.925388000	356.269607000
H	-35.780490000	35.179674000	356.155390000
H	-36.799935000	35.751094000	357.190274000
O	-34.618467000	42.817191000	367.548377000
H	-35.095516000	43.614267000	367.183915000
H	-35.325726000	42.256506000	367.976084000
O	-33.954934000	39.827442000	359.990197000
H	-34.480230000	38.981113000	359.867347000
H	-34.625251000	40.550472000	359.894104000
O	-34.977240000	38.057210000	357.039234000
H	-35.539883000	38.865905000	357.154574000
H	-35.566326000	37.331267000	356.656434000
O	-33.138176000	43.798124000	363.827875000
H	-32.978040000	43.296464000	362.990423000
H	-33.041191000	43.097695000	364.548273000
O	-30.398035000	37.328967000	368.289447000
H	-30.199933000	36.905259000	367.427632000
H	-31.069687000	37.990636000	368.000159000
O	-33.964576000	46.116188000	364.828873000
H	-33.184371000	46.310104000	365.382882000
H	-33.684397000	45.281096000	364.324255000
H	-37.037459000	41.043942000	367.816677000
H	-37.213711000	41.877019000	369.129996000
O	-32.850183000	41.946201000	365.737182000
H	-33.028569000	41.063710000	365.317937000
H	-33.594720000	42.130924000	366.387323000
O	-36.591232000	41.269820000	368.688408000
H	-32.025280000	34.769662000	361.465806000

Open O4H W1[H_2O] W2[OH]

Ca	-32.663599000	39.789801000	362.028741000
O	-33.187084000	39.554758000	364.369283000
O	-31.672606000	37.733659000	363.235388000
O	-33.631152000	37.114770000	364.730364000
O	-32.243689000	35.685014000	361.501785000
O	-33.903656000	37.442722000	361.865998000
Mn	-34.779219000	38.642760000	364.655163000
Mn	-32.070735000	38.200156000	364.941937000
Mn	-32.951946000	36.433896000	363.056567000

Mn	-33.704769000	36.327879000	360.395118000
C	-30.033546000	34.448867000	365.303944000
C	-31.043696000	35.488534000	364.977219000
O	-31.019188000	36.617124000	365.582933000
O	-31.868055000	35.161782000	364.057509000
H	-29.241670000	34.494808000	364.532920000
H	-30.485764000	33.445273000	365.267378000
H	-29.564637000	34.619503000	366.285264000
C	-32.796152000	30.220033000	361.294719000
C	-33.434689000	31.330047000	360.440915000
O	-32.753831000	32.436445000	360.294303000
O	-34.563225000	31.139418000	359.918737000
H	-33.431731000	29.322177000	361.299852000
H	-32.660448000	30.579061000	362.331024000
H	-31.796824000	29.970434000	360.897041000
C	-29.073591000	44.563795000	354.927778000
C	-30.026823000	43.367699000	355.034116000
C	-30.959666000	43.371495000	356.235598000
C	-31.743351000	42.231651000	356.531864000
C	-31.097873000	44.485000000	357.088784000
C	-32.606042000	42.191577000	357.637524000
C	-31.958386000	44.462932000	358.197749000
C	-32.713982000	43.306951000	358.506125000
O	-33.496700000	43.255077000	359.612956000
H	-29.622319000	45.516354000	354.814300000
H	-30.636581000	43.306724000	354.109168000
H	-29.437202000	42.429342000	355.052049000
H	-31.672075000	41.350866000	355.877556000
H	-30.517199000	45.395688000	356.898707000
H	-33.203191000	41.296527000	357.856755000
H	-32.027037000	45.344299000	358.845275000
H	-33.535627000	44.499953000	360.384543000
H	-28.432131000	44.650811000	355.824429000
H	-28.412489000	44.455892000	354.049962000
C	-29.253922000	43.004351000	359.721418000
C	-29.687260000	44.200649000	360.532226000
N	-29.150949000	45.392827000	360.199131000
O	-30.502165000	44.096744000	361.483350000
H	-28.492616000	43.247014000	358.964210000
H	-30.138001000	42.583021000	359.211195000
H	-28.515491000	45.491208000	359.415313000
H	-29.421735000	46.231070000	360.705587000
H	-28.861111000	42.233191000	360.405931000
C	-30.088214000	34.401171000	356.344816000
C	-31.097302000	35.486685000	356.653758000
O	-32.310144000	35.196555000	356.878743000
H	-30.402799000	33.889267000	355.418338000
H	-30.104901000	33.653127000	357.156066000
H	-29.063351000	34.784357000	356.217207000
N	-30.657410000	36.757285000	356.656392000
C	-31.497340000	37.952711000	356.786156000
C	-31.165621000	38.801997000	358.017284000
C	-31.628213000	38.229556000	359.361796000
O	-31.229766000	38.737413000	360.437467000
O	-32.472785000	37.245134000	359.262000000
H	-29.672578000	36.903881000	356.445505000
H	-32.550693000	37.632731000	356.819618000

H	-30.087330000	39.032158000	358.091523000
H	-31.680286000	39.777560000	357.940040000
H	-31.348927000	38.563682000	355.877242000
C	-36.293781000	42.469505000	363.932042000
C	-35.627176000	41.170867000	363.554699000
O	-34.915053000	41.023395000	362.539992000
O	-35.873983000	40.197460000	364.404741000
H	-36.008236000	42.733900000	364.964457000
H	-37.391495000	42.357957000	363.881241000
H	-35.976075000	43.278436000	363.258288000
C	-36.037814000	48.310983000	361.463650000
C	-34.991554000	47.268070000	361.326429000
C	-35.022280000	46.031275000	360.713828000
N	-33.710408000	47.402068000	361.843112000
C	-32.993593000	46.293846000	361.565346000
N	-33.780139000	45.455239000	360.879710000
H	-36.940788000	48.011197000	360.908536000
H	-35.694079000	49.282772000	361.066482000
H	-35.820813000	45.528310000	360.169101000
H	-31.963199000	46.088807000	361.856894000
H	-36.322359000	48.466051000	362.520573000
C	-34.720683000	51.637782000	364.203879000
C	-34.292556000	50.193711000	363.974067000
N	-34.838127000	49.248135000	364.770476000
O	-33.472169000	49.892356000	363.068721000
H	-35.411784000	51.749788000	365.053911000
H	-35.206295000	52.011570000	363.285762000
H	-34.570282000	48.264788000	364.669341000
H	-35.496021000	49.491518000	365.503561000
H	-33.820159000	52.250955000	364.378509000
H	-33.408595000	48.249369000	362.377877000
C	-40.681661000	40.131028000	367.699860000
C	-40.752553000	38.762851000	368.310738000
O	-40.341653000	37.716349000	367.735305000
H	-41.369112000	40.175611000	366.835581000
N	-41.301349000	38.684005000	369.531737000
C	-41.433296000	37.412209000	370.206975000
H	-41.649428000	39.526613000	369.977893000
H	-40.447187000	36.931820000	370.334156000
C	-39.851593000	36.488341000	364.048019000
C	-38.556884000	36.960890000	364.593124000
C	-37.310909000	37.129953000	364.012358000
N	-38.412874000	37.347456000	365.916758000
C	-37.143660000	37.738146000	366.123761000
N	-36.442867000	37.613583000	364.981880000
H	-39.759463000	36.276268000	362.970733000
H	-40.642962000	37.248460000	364.180799000
H	-36.994006000	36.976886000	362.979871000
H	-36.755997000	38.135511000	367.059992000
H	-40.190931000	35.565060000	364.552396000
H	-40.951770000	40.928154000	368.410452000
H	-39.662586000	40.301173000	367.313290000
H	-41.881157000	37.579636000	371.197858000
H	-42.078015000	36.722763000	369.633200000
H	-39.196902000	37.473960000	366.618171000
C	-36.210088000	33.753083000	362.748233000
C	-35.115682000	34.694177000	362.352362000

O	-34.334690000	35.076054000	363.288236000
O	-35.036802000	35.077644000	361.136170000
H	-35.801262000	32.955103000	363.388993000
H	-36.705782000	33.322046000	361.866134000
H	-36.952716000	34.315314000	363.343596000
C	-33.613534000	34.102019000	370.372090000
C	-33.933859000	34.606743000	368.998348000
C	-33.260074000	35.457643000	368.136118000
N	-35.092672000	34.242733000	368.316297000
C	-35.135499000	34.829296000	367.107197000
N	-34.027432000	35.569005000	366.984978000
H	-34.399982000	34.390754000	371.091867000
H	-32.656166000	34.527317000	370.710514000
H	-32.309946000	35.987742000	368.255397000
H	-35.922672000	34.716378000	366.363727000
H	-33.817057000	36.141548000	366.131968000
H	-33.530026000	33.000648000	370.377924000
H	-35.814096000	33.619214000	368.673688000
C	-33.579444000	39.662687000	368.687086000
C	-33.601112000	39.042857000	367.329340000
O	-32.490477000	38.562508000	366.866887000
O	-34.693383000	39.039200000	366.688973000
H	-34.599742000	39.894781000	369.024066000
H	-32.998724000	40.601320000	368.624410000
H	-33.063225000	39.008613000	369.410747000
C	-29.204110000	41.144943000	365.018420000
C	-30.179613000	40.211799000	364.376330000
O	-30.580395000	40.350084000	363.199896000
O	-30.569401000	39.270047000	365.202564000
H	-29.784406000	41.842167000	365.651392000
H	-28.505054000	40.601909000	365.674220000
H	-28.657722000	41.730066000	364.262655000
C	-25.798943000	37.259595000	362.310142000
N	-27.048162000	36.552512000	362.015391000
C	-28.271727000	37.110439000	362.008606000
N	-28.426925000	38.415105000	362.286164000
N	-29.354851000	36.364566000	361.737371000
H	-25.831480000	37.716307000	363.315307000
H	-25.589723000	38.040781000	361.556675000
H	-26.988271000	35.572659000	361.758433000
H	-27.621598000	39.024686000	362.364925000
H	-29.349602000	38.848782000	362.244675000
H	-29.288164000	35.361368000	361.595080000
H	-30.290342000	36.774410000	361.768821000
H	-24.983648000	36.523213000	362.287063000
O	-30.995474000	33.510580000	361.745797000
H	-31.641281000	32.899233000	361.203246000
H	-31.163409000	33.305947000	362.686522000
O	-35.647320000	41.834152000	359.861906000
H	-34.834780000	42.436262000	359.688991000
H	-35.652227000	41.722784000	360.841592000
O	-33.429911000	34.741155000	359.156606000
H	-33.142507000	33.843429000	359.578378000
H	-32.891959000	34.935093000	358.311556000
O	-32.300166000	42.100148000	361.614316000
H	-31.461557000	42.647600000	361.632739000
H	-32.782642000	42.479125000	360.802320000

O	-35.772156000	45.104863000	366.588154000
H	-35.132018000	45.493105000	365.913939000
H	-36.615926000	45.025001000	366.107465000
O	-35.050867000	37.121453000	359.488535000
H	-34.835083000	37.430176000	358.509837000
O	-35.601073000	33.171547000	358.449778000
H	-34.947961000	33.860693000	358.727286000
H	-35.278747000	32.374508000	358.982056000
O	-37.738232000	40.783133000	366.336379000
H	-38.137565000	41.603980000	365.994388000
H	-37.046717000	40.541802000	365.660474000
O	-37.209981000	35.493607000	358.766335000
H	-36.490823000	36.044421000	359.180463000
H	-36.854652000	34.571774000	358.823226000
O	-34.676559000	33.903767000	355.910726000
H	-34.984783000	33.450051000	356.742156000
H	-33.782564000	34.249913000	356.143120000
O	-36.312811000	40.346981000	357.551822000
H	-36.146733000	40.858368000	358.384995000
H	-36.151444000	40.994159000	356.840348000
O	-36.379556000	36.073351000	356.178889000
H	-35.751869000	35.312192000	356.004304000
H	-36.782624000	35.836895000	357.064233000
O	-34.591415000	42.805692000	367.534544000
H	-35.074449000	43.603882000	367.179071000
H	-35.290964000	42.243057000	367.972451000
O	-33.969084000	39.705849000	360.009964000
H	-34.480803000	38.864662000	359.894193000
H	-34.633577000	40.437868000	359.878667000
O	-34.828167000	38.027751000	357.107873000
H	-35.361134000	38.863156000	357.224730000
H	-35.441258000	37.352349000	356.659026000
O	-33.154959000	43.801548000	363.803823000
H	-32.986188000	43.302450000	362.968559000
H	-33.049122000	43.104860000	364.525630000
O	-30.448112000	37.328199000	368.370458000
H	-30.134846000	36.896181000	367.550575000
H	-31.099523000	37.969507000	368.001693000
O	-33.980730000	46.121393000	364.826323000
H	-33.196663000	46.319221000	365.373735000
H	-33.700528000	45.293341000	364.314842000
H	-37.031070000	41.066373000	367.849079000
H	-37.143093000	41.869700000	369.189875000
O	-32.839991000	41.954796000	365.717537000
H	-33.033052000	41.076681000	365.301785000
H	-33.572925000	42.143472000	366.383624000
O	-36.548735000	41.265907000	368.706724000
H	-31.782420000	34.738925000	361.591363000

Open O4 W1[OH] W2[H_2O]

Ca	-32.672758000	39.757775000	362.075604000
O	-33.157960000	39.565417000	364.400482000

O	-31.657156000	37.755875000	363.242255000
O	-33.587251000	37.113601000	364.813310000
O	-32.422359000	35.794855000	361.442924000
O	-34.058226000	37.639081000	362.151215000
Mn	-34.736774000	38.614117000	364.590595000
Mn	-32.036798000	38.188639000	364.950728000
Mn	-33.009598000	36.502528000	363.045978000
Mn	-33.705411000	36.275072000	360.294718000
C	-30.033546000	34.448867000	365.303944000
C	-31.041511000	35.480475000	364.927674000
O	-31.011287000	36.603183000	365.570776000
O	-31.833645000	35.187304000	363.984838000
H	-30.369112000	33.446716000	364.999001000
H	-29.810412000	34.470685000	366.383063000
H	-29.095471000	34.679318000	364.764702000
C	-32.796152000	30.220033000	361.294719000
C	-33.378629000	31.202572000	360.266562000
O	-32.567247000	31.907118000	359.591874000
O	-34.659180000	31.219845000	360.129881000
H	-33.572104000	29.801181000	361.956519000
H	-32.021880000	30.724826000	361.899184000
H	-32.302213000	29.389323000	360.757439000
C	-29.073591000	44.563795000	354.927778000
C	-30.044742000	43.381324000	355.024539000
C	-30.970202000	43.386152000	356.231594000
C	-31.742693000	42.242228000	356.541461000
C	-31.111182000	44.504610000	357.077812000
C	-32.597440000	42.202793000	357.653030000
C	-31.965129000	44.484312000	358.191806000
C	-32.709856000	43.324500000	358.513316000
O	-33.484641000	43.273084000	359.623678000
H	-29.607518000	45.525725000	354.823083000
H	-30.659593000	43.341177000	354.101619000
H	-29.469826000	42.433870000	355.028392000
H	-31.665843000	41.356192000	355.894842000
H	-30.537284000	45.417652000	356.878351000
H	-33.179974000	41.302052000	357.888267000
H	-32.035361000	45.368557000	358.835231000
H	-33.522976000	44.498634000	360.392305000
H	-28.430977000	44.632854000	355.825197000
H	-28.414394000	44.453968000	354.048674000
C	-29.253922000	43.004351000	359.721419000
C	-29.682064000	44.215335000	360.513642000
N	-29.147177000	45.401677000	360.152043000
O	-30.489222000	44.130114000	361.471553000
H	-28.475102000	43.228271000	358.976233000
H	-30.136378000	42.594836000	359.198414000
H	-28.527805000	45.484312000	359.353817000
H	-29.425653000	46.251503000	360.634176000
H	-28.889135000	42.230995000	360.418550000
C	-30.088215000	34.401171000	356.344816000
C	-31.098555000	35.508319000	356.568956000
O	-32.332630000	35.264557000	356.650898000
H	-30.364643000	33.850333000	355.429492000
H	-30.153081000	33.694974000	357.191292000
H	-29.054150000	34.770097000	356.253410000
N	-30.622403000	36.766330000	356.675697000

C	-31.458874000	37.964220000	356.772542000
C	-31.165621000	38.801997000	358.017283000
C	-31.681838000	38.262474000	359.344735000
O	-31.250304000	38.709791000	360.430966000
O	-32.626894000	37.381248000	359.199820000
H	-29.618392000	36.899049000	356.581052000
H	-32.512755000	37.644244000	356.767742000
H	-30.085910000	39.012062000	358.128897000
H	-31.647969000	39.791474000	357.910316000
H	-31.278739000	38.581821000	355.872820000
C	-36.293781000	42.469506000	363.932042000
C	-35.615487000	41.178168000	363.539628000
O	-34.896351000	41.069273000	362.524018000
O	-35.855708000	40.183490000	364.360686000
H	-36.033216000	42.711448000	364.976407000
H	-37.390311000	42.361390000	363.850393000
H	-35.959342000	43.292385000	363.283528000
C	-36.037814000	48.310983000	361.463650000
C	-34.987430000	47.270727000	361.331420000
C	-35.014623000	46.030322000	360.725796000
N	-33.705895000	47.410976000	361.845894000
C	-32.986751000	46.302385000	361.573024000
N	-33.770911000	45.458157000	360.892805000
H	-36.939428000	48.005260000	360.909480000
H	-35.697882000	49.282441000	361.062224000
H	-35.812755000	45.521451000	360.185856000
H	-31.955074000	46.101047000	361.863219000
H	-36.323611000	48.469853000	362.519749000
C	-34.720683000	51.637782000	364.203879000
C	-34.282912000	50.196768000	363.970704000
N	-34.822642000	49.244701000	364.763561000
O	-33.459911000	49.904783000	363.065031000
H	-35.407334000	51.744390000	365.058283000
H	-35.215266000	52.007632000	363.288944000
H	-34.551171000	48.262538000	364.658194000
H	-35.484381000	49.480811000	365.495555000
H	-33.823810000	52.257768000	364.372760000
H	-33.403557000	48.260889000	362.375178000
C	-40.681662000	40.131027000	367.699860000
C	-40.738913000	38.762418000	368.312989000
O	-40.304620000	37.722294000	367.747154000
H	-41.346476000	40.158542000	366.817411000
N	-41.305052000	38.682115000	369.527737000
C	-41.433296000	37.412209000	370.206975000
H	-41.669227000	39.522443000	369.964981000
H	-40.443731000	36.945400000	370.356954000
C	-39.851593000	36.488341000	364.048019000
C	-38.541107000	36.948827000	364.570461000
C	-37.300971000	37.110381000	363.973800000
N	-38.375227000	37.330232000	365.893264000
C	-37.098271000	37.711439000	366.081673000
N	-36.416388000	37.587010000	364.930558000
H	-39.780929000	36.276760000	362.968847000
H	-40.634496000	37.254810000	364.195033000
H	-36.988527000	36.945604000	362.941849000
H	-36.693346000	38.108009000	367.010866000
H	-40.190684000	35.566731000	364.555984000

H	-40.988294000	40.924015000	368.400273000
H	-39.657591000	40.324209000	367.338319000
H	-41.903598000	37.578114000	371.187713000
H	-42.056368000	36.710903000	369.623587000
H	-39.145257000	37.463007000	366.600087000
C	-36.210088000	33.753083000	362.748233000
C	-35.163440000	34.753057000	362.368247000
O	-34.372732000	35.126161000	363.296273000
O	-35.125234000	35.144765000	361.151246000
H	-35.928618000	32.777039000	362.309003000
H	-37.185644000	34.032424000	362.317143000
H	-36.286658000	33.657444000	363.840435000
C	-33.613534000	34.102019000	370.372090000
C	-33.920024000	34.615181000	368.998158000
C	-33.237250000	35.470204000	368.146962000
N	-35.072878000	34.257357000	368.302888000
C	-35.100045000	34.854027000	367.096560000
N	-33.990908000	35.591607000	366.989304000
H	-34.405909000	34.386766000	371.087138000
H	-32.659075000	34.524766000	370.722111000
H	-32.285842000	35.995447000	368.275259000
H	-35.880407000	34.750756000	366.344665000
H	-33.772642000	36.181477000	366.133796000
H	-33.529805000	33.000510000	370.373471000
H	-35.798105000	33.631759000	368.647539000
C	-33.579444000	39.662687000	368.687086000
C	-33.583923000	39.040773000	367.325002000
O	-32.455026000	38.584179000	366.884295000
O	-34.671061000	39.019496000	366.678413000
H	-34.605498000	39.863575000	369.027329000
H	-33.030912000	40.620253000	368.623923000
H	-33.043224000	39.024596000	369.410439000
C	-29.204110000	41.144943000	365.018420000
C	-30.160269000	40.200557000	364.372378000
O	-30.561830000	40.348065000	363.196687000
O	-30.523086000	39.252071000	365.196480000
H	-29.799511000	41.836290000	365.644145000
H	-28.503192000	40.614936000	365.682991000
H	-28.657599000	41.739344000	364.269300000
C	-25.798943000	37.259595000	362.310142000
N	-27.047215000	36.519218000	362.069344000
C	-28.286030000	37.052915000	362.044751000
N	-28.456907000	38.368589000	362.267799000
N	-29.360108000	36.294436000	361.778974000
H	-25.764529000	37.669082000	363.336237000
H	-25.681410000	38.081337000	361.581669000
H	-26.980299000	35.515723000	361.933921000
H	-27.702441000	38.924581000	362.652595000
H	-29.396004000	38.769869000	362.289265000
H	-29.296361000	35.327416000	361.464271000
H	-30.298901000	36.696566000	361.850789000
H	-24.965006000	36.555105000	362.184475000
O	-30.791768000	33.661196000	360.794566000
H	-31.378601000	33.000740000	360.328314000
H	-31.437349000	34.369976000	361.042838000
O	-35.672150000	41.909320000	359.892181000
H	-34.849876000	42.490965000	359.706134000

H	-35.638748000	41.780164000	360.871240000
O	-33.526773000	34.988393000	359.057068000
H	-33.021141000	35.258600000	358.237601000
O	-32.291173000	42.103132000	361.642363000
H	-31.449322000	42.638762000	361.655777000
H	-32.767157000	42.475712000	360.829624000
O	-35.832050000	45.101150000	366.532994000
H	-35.167425000	45.479946000	365.876311000
H	-36.651276000	44.993214000	366.016699000
O	-35.368797000	37.253956000	359.356842000
H	-35.119519000	37.560976000	358.386554000
O	-35.362653000	33.194807000	358.577312000
H	-34.615032000	33.823029000	358.830674000
H	-35.121438000	32.372561000	359.164066000
O	-37.741796000	40.714950000	366.257889000
H	-38.128014000	41.539883000	365.911401000
H	-37.030052000	40.478441000	365.596110000
O	-37.021395000	35.237728000	358.672553000
H	-36.060185000	36.527463000	359.224558000
H	-36.481630000	34.372716000	358.764316000
O	-34.620826000	33.804370000	355.993581000
H	-34.887071000	33.422194000	356.880918000
H	-33.724214000	34.185251000	356.166532000
O	-36.345336000	40.501789000	357.479475000
H	-36.170580000	40.938546000	358.351148000
H	-36.085852000	41.176529000	356.824925000
O	-36.314016000	35.968710000	356.051993000
H	-35.650723000	35.217338000	355.952542000
H	-36.769057000	35.723918000	356.897709000
O	-34.651605000	42.809326000	367.538767000
H	-35.136951000	43.597554000	367.168117000
H	-35.353543000	42.232821000	367.953269000
O	-33.986899000	39.775890000	360.011803000
H	-34.543769000	38.967907000	359.971844000
H	-34.639250000	40.521514000	359.907468000
O	-34.948201000	38.103544000	356.978843000
H	-35.495547000	38.930810000	357.049113000
H	-35.501343000	37.399168000	356.511341000
O	-33.123294000	43.792371000	363.825823000
H	-32.971114000	43.286032000	362.990186000
H	-33.042294000	43.090330000	364.548678000
O	-30.379786000	37.293205000	368.263879000
H	-30.233070000	36.880092000	367.384703000
H	-31.046785000	37.975011000	368.013557000
O	-33.982652000	46.100254000	364.825679000
H	-33.212980000	46.286948000	365.396879000
H	-33.693463000	45.268827000	364.320126000
H	-37.063057000	41.011136000	367.778474000
H	-37.253056000	41.845235000	369.090055000
O	-32.860912000	41.946254000	365.736138000
H	-33.032792000	41.060378000	365.317244000
H	-33.607099000	42.125255000	366.384686000
O	-36.625341000	41.239700000	368.653706000
H	-37.858065000	35.084243000	359.146937000

Closed O4 W1[H_2O] W2[OH]

Ca	-32.695651000	39.789304000	362.091744000
O	-33.084125000	39.592907000	364.503040000
O	-31.743584000	37.776703000	363.305274000
O	-33.552934000	37.155104000	365.046571000
O	-32.600823000	35.972092000	361.642689000
O	-34.382952000	37.960169000	362.866569000
Mn	-34.639065000	38.603000000	364.578162000
Mn	-31.977647000	38.213742000	365.034802000
Mn	-33.139416000	36.567865000	363.224081000
Mn	-33.805111000	36.338326000	360.301749000
C	-30.033546000	34.448867000	365.303944000
C	-31.036706000	35.504358000	364.952599000
O	-30.945866000	36.627084000	365.588597000
O	-31.911046000	35.223029000	364.074797000
H	-29.880448000	33.780116000	364.442633000
H	-30.423399000	33.846356000	366.145534000
H	-29.083033000	34.905477000	365.621039000
C	-32.796152000	30.220033000	361.294719000
C	-33.404237000	31.423841000	360.556712000
O	-32.594240000	32.378643000	360.226557000
O	-34.641808000	31.400980000	360.285042000
H	-33.580107000	29.619329000	361.781811000
H	-32.052409000	30.552742000	362.038862000
H	-32.269690000	29.584369000	360.558858000
C	-29.073591000	44.563795000	354.927778000
C	-30.043834000	43.380424000	355.028440000
C	-30.967031000	43.383483000	356.237608000
C	-31.744224000	42.241253000	356.543145000
C	-31.101985000	44.498156000	357.089657000
C	-32.596411000	42.199580000	357.656586000
C	-31.952819000	44.475124000	358.206205000
C	-32.700416000	43.316124000	358.524201000
O	-33.471046000	43.259797000	359.637549000
H	-29.608737000	45.525363000	354.825827000
H	-30.660932000	43.338937000	354.107125000
H	-29.467792000	42.433599000	355.031166000
H	-31.673811000	41.358384000	355.891592000
H	-30.525763000	45.410423000	356.892972000
H	-33.186296000	41.302105000	357.886228000
H	-32.017479000	45.356474000	358.854185000
H	-33.529104000	44.488956000	360.389306000
H	-28.426999000	44.633094000	355.822337000
H	-28.418356000	44.454288000	354.045703000
C	-29.253922000	43.004351000	359.721419000
C	-29.666151000	44.214390000	360.522914000
N	-29.132445000	45.400020000	360.158545000
O	-30.459093000	44.127769000	361.493074000
H	-28.502433000	43.231786000	358.950048000
H	-30.148534000	42.582977000	359.229872000
H	-28.526308000	45.484318000	359.350233000
H	-29.400210000	46.248620000	360.648975000
H	-28.857654000	42.238055000	360.409261000
C	-30.088215000	34.401171000	356.344816000

C	-31.116120000	35.484517000	356.616964000
O	-32.334297000	35.197647000	356.777572000
H	-30.363786000	33.886360000	355.407903000
H	-30.131414000	33.654789000	357.156848000
H	-29.060166000	34.787985000	356.257959000
N	-30.664060000	36.754366000	356.661797000
C	-31.495730000	37.955671000	356.782783000
C	-31.165621000	38.801997000	358.017283000
C	-31.676881000	38.239832000	359.347426000
O	-31.321602000	38.770303000	360.433564000
O	-32.504574000	37.255589000	359.223818000
H	-29.668567000	36.894166000	356.505952000
H	-32.550436000	37.639755000	356.815057000
H	-30.082142000	39.003112000	358.108767000
H	-31.645774000	39.792656000	357.922116000
H	-31.342030000	38.564653000	355.872430000
C	-36.293781000	42.469506000	363.932042000
C	-35.538273000	41.205793000	363.568791000
O	-34.798471000	41.135255000	362.566896000
O	-35.733373000	40.200766000	364.393619000
H	-36.070809000	42.737088000	364.978826000
H	-37.380808000	42.306289000	363.823689000
H	-35.977793000	43.295182000	363.278359000
C	-36.037814000	48.310983000	361.463650000
C	-34.990103000	47.268157000	361.328286000
C	-35.021519000	46.026266000	360.725428000
N	-33.705400000	47.408681000	361.834724000
C	-32.988755000	46.298744000	361.560066000
N	-33.777389000	45.453171000	360.886571000
H	-36.943378000	48.005276000	360.915959000
H	-35.698263000	49.280393000	361.057060000
H	-35.823055000	45.517166000	360.190733000
H	-31.955334000	46.098353000	361.845145000
H	-36.316817000	48.473709000	362.520970000
C	-34.720683000	51.637782000	364.203879000
C	-34.279442000	50.198297000	363.967012000
N	-34.809592000	49.244719000	364.764531000
O	-33.462530000	49.908772000	363.055120000
H	-35.404431000	51.741079000	365.061027000
H	-35.219458000	52.007949000	363.291365000
H	-34.533696000	48.263489000	364.658583000
H	-35.466200000	49.479745000	365.501450000
H	-33.824919000	52.260021000	364.370626000
H	-33.400381000	48.258561000	362.361392000
C	-40.681662000	40.131027000	367.699860000
C	-40.724960000	38.763980000	368.318625000
O	-40.260662000	37.729796000	367.766195000
H	-41.347194000	40.149103000	366.817697000
N	-41.312081000	38.680221000	369.523212000
C	-41.433296000	37.412209000	370.206975000
H	-41.699317000	39.516568000	369.948062000
H	-40.439566000	36.962047000	370.378809000
C	-39.851593000	36.488341000	364.048019000
C	-38.527808000	36.978323000	364.553198000
C	-37.289862000	37.167453000	363.950529000
N	-38.348698000	37.349167000	365.880929000
C	-37.073850000	37.743254000	366.063482000

N	-36.396792000	37.642525000	364.906800000
H	-39.801077000	36.306765000	362.962496000
H	-40.648340000	37.229872000	364.237979000
H	-36.981664000	37.023848000	362.914573000
H	-36.670703000	38.128951000	366.998057000
H	-40.143740000	35.544787000	364.543692000
H	-40.993362000	40.924797000	368.396985000
H	-39.658493000	40.327837000	367.337494000
H	-41.925046000	37.575768000	371.177526000
H	-42.033778000	36.697799000	369.615805000
H	-39.111131000	37.471597000	366.597070000
C	-36.210088000	33.753083000	362.748233000
C	-35.248529000	34.858062000	362.450719000
O	-34.510617000	35.228113000	363.432203000
O	-35.217581000	35.340657000	361.270763000
H	-35.837334000	32.835955000	362.252102000
H	-37.197641000	33.974805000	362.310708000
H	-36.295965000	33.578364000	363.829842000
C	-33.613534000	34.102019000	370.372090000
C	-33.993441000	34.594996000	369.019906000
C	-33.351530000	35.456072000	368.148711000
N	-35.167808000	34.224051000	368.372359000
C	-35.246859000	34.822871000	367.168556000
N	-34.149361000	35.569890000	367.025039000
H	-34.369055000	34.383616000	371.127618000
H	-32.647114000	34.538395000	370.668661000
H	-32.398675000	35.985850000	368.246874000
H	-36.053744000	34.712194000	366.446220000
H	-33.933729000	36.184378000	366.186022000
H	-33.516158000	33.001413000	370.384171000
H	-35.871602000	33.589698000	368.744508000
C	-33.579444000	39.662687000	368.687086000
C	-33.542853000	39.082618000	367.304537000
O	-32.389989000	38.643398000	366.928223000
O	-34.605253000	39.077953000	366.593877000
H	-34.606211000	39.919425000	368.987424000
H	-32.962928000	40.579273000	368.685700000
H	-33.127097000	38.957343000	369.404728000
C	-29.204110000	41.144943000	365.018420000
C	-30.129568000	40.189110000	364.363086000
O	-30.559806000	40.333274000	363.200298000
O	-30.443379000	39.220426000	365.189336000
H	-29.817185000	41.795335000	365.670896000
H	-28.476085000	40.623716000	365.660129000
H	-28.687982000	41.781778000	364.282485000
C	-25.798943000	37.259595000	362.310142000
N	-27.097433000	36.589514000	362.162504000
C	-28.293616000	37.209228000	362.095110000
N	-28.354878000	38.554637000	362.158199000
N	-29.424165000	36.511732000	361.933824000
H	-25.700370000	37.741656000	363.300315000
H	-25.655898000	38.014780000	361.517478000
H	-27.110287000	35.575167000	362.186698000
H	-27.568209000	39.084781000	362.515209000
H	-29.263196000	39.020690000	362.143781000
H	-29.451385000	35.512210000	361.707939000
H	-30.330699000	36.982229000	362.034507000

H	-25.014572000	36.496007000	362.211678000
O	-30.693303000	33.964588000	361.491208000
H	-31.197963000	33.239733000	361.040069000
H	-31.411125000	34.634836000	361.642787000
O	-35.658330000	41.886619000	359.936861000
H	-34.832053000	42.452999000	359.732181000
H	-35.606102000	41.765449000	360.914774000
O	-33.431860000	34.565080000	359.146434000
H	-33.080313000	33.678024000	359.581123000
H	-32.908716000	34.768251000	358.318683000
O	-32.267375000	42.115394000	361.658535000
H	-31.423275000	42.648777000	361.665110000
H	-32.746662000	42.483963000	360.843856000
O	-35.771661000	45.150196000	366.555862000
H	-35.111082000	45.526555000	365.894574000
H	-36.595405000	45.045538000	366.046042000
O	-35.164142000	37.244152000	359.393516000
H	-34.921122000	37.523211000	358.448710000
O	-35.673412000	33.235375000	358.552047000
H	-34.929938000	33.876088000	358.744701000
H	-35.394409000	32.491908000	359.174271000
O	-37.694689000	40.709080000	366.261570000
H	-38.105760000	41.507318000	365.882230000
H	-36.964114000	40.479631000	365.622103000
O	-37.236432000	35.584563000	358.738400000
H	-36.520677000	36.185974000	359.105650000
H	-36.841320000	34.679532000	358.833971000
O	-34.750517000	33.895562000	355.966126000
H	-35.058986000	33.467223000	356.809265000
H	-33.857184000	34.253146000	356.185009000
O	-36.380919000	40.491469000	357.575721000
H	-36.188135000	40.943569000	358.436488000
H	-36.186554000	41.172420000	356.905407000
O	-36.462180000	36.097632000	356.144539000
H	-35.826724000	35.339556000	356.002100000
H	-36.834593000	35.900859000	357.056340000
O	-34.609061000	42.860941000	367.570158000
H	-35.087133000	43.652443000	367.195325000
H	-35.319297000	42.283102000	367.969171000
O	-34.061411000	39.650069000	360.116468000
H	-34.565513000	38.816349000	359.881765000
H	-34.698313000	40.396135000	359.968229000
O	-34.884561000	38.173771000	356.902481000
H	-35.439894000	38.973377000	357.095358000
H	-35.508063000	37.474067000	356.531731000
O	-33.144629000	43.814911000	363.826767000
H	-32.965325000	43.308245000	362.996804000
H	-33.032157000	43.130236000	364.558835000
O	-30.438635000	37.162742000	368.355432000
H	-30.277649000	36.796672000	367.458433000
H	-31.033567000	37.913611000	368.133844000
O	-33.933992000	46.145508000	364.831037000
H	-33.154494000	46.336673000	365.387197000
H	-33.654513000	45.312538000	364.323647000
H	-37.035800000	41.051848000	367.778537000
H	-37.213272000	41.873920000	369.100809000
O	-32.813580000	42.010970000	365.782120000

H	-32.985732000	41.116678000	365.387515000
H	-33.558955000	42.200120000	366.432571000
O	-36.587150000	41.278375000	368.648748000
